# Pre-Conceptional Exposure to Glyphosate Affects the Maternal Hepatic and Ovarian Proteome

**DOI:** 10.1093/toxsci/kfac098

**Published:** 2022-09-29

**Authors:** Gulnara Novbatova, Kelsey Timme, Andrew Severin, Maryam Sayadi, Aileen F Keating

**Affiliations:** Department of Animal Science, Iowa State University, Ames, Iowa 50011, USA; Department of Animal Science, Iowa State University, Ames, Iowa 50011, USA; Department of Animal Science, Iowa State University, Ames, Iowa 50011, USA; Department of Animal Science, Iowa State University, Ames, Iowa 50011, USA; Department of Animal Science, Iowa State University, Ames, Iowa 50011, USA

**Keywords:** glyphosate, ovary, proteome, pregnancy

## Abstract

Exposure to glyphosate (GLY), a commonly used herbicide, is supported by urinary detection and associated with shortened gestation in women. This study tested the hypothesis that chronic low-dose pre-conceptional GLY exposure would affect maternal ovarian function mid- and post-gestation. Mice (C57BL/6; *n* = 40) were exposed *per os* to saline vehicle control (CT; *n* = 20) or GLY (2 mg/kg; *n* = 20) daily for 10 weeks starting at 7 weeks of age. Post-exposure, females were impregnated and euthanized at gestation day 14 (GD14) or post-weaning (PW). Pregnancy success was reduced from 75% to 55% by GLY exposure. No treatment effect (*p *>* *.05) on body weight, maternal serum 17β-estradiol, or litter size was noted. Ovarian weight was unaffected or reduced (*p < *.05) by GLY in GD14 and PW dams, respectively. Exposure to GLY decreased (*p *<* *.05) PW ovarian secondary follicle number with no other follicle composition impacts. Protein abundance analysis by LC-MS/MS identified that GLY altered (*p *<* *.05) 26 ovarian and 41 hepatic proteins in GD14 dams and 39 hepatic proteins in PW dams. In GD14 dams, GLY increased ovarian protein abundance of SEC16A (*p *<* *.05; 29-fold) and hepatic RPS27L and GM4952 (*p *<* *.05; ∼4-fold). In both GD14 and PW dams, GLY exposure increased (*p *<* *.05) hepatic RPS4 and decreased (*p *<* *.05) ECHDC3. Pathway analysis using DAVID identified 10 GLY hepatic pathway targets with FDR ≤ 0.07 in GD14 dams.

Impact statement Pre-conceptional GLY exposure affects the maternal ovarian and hepatic proteome.

Ovotoxicant exposures compromise the ovary in a variety of ways including but not limited to, depletion of primordial germ cells (Borman *et al.*, 2000; Hoyer and Keating, 2014; Mattison and Schulman, 1980), primordial follicle growth hyperactivation (Keating *et al.*, 2011), induction of DNA damage (Ganesan *et al.*, 2013), and alteration to follicular growth and viability (Hoyer, 2001) at each stage of folliculogenesis. In addition, ovotoxicants can affect steroidogenesis and result in premature follicular loss (Krarup, 1969) and early menopause as a consequence (Hoyer, 2001; Hoyer and Keating, 2014). Additionally, negative impacts on pre- and peri-pubertal sexual maturation in exposed animals are documented (Hatch *et al.*, 2011; Özen and Darcan, 2011; Yang *et al.*, 2016). Further, ovotoxicant exposure during fetal development can harm offspring germ cell number and quality (Brieño-Enríquez *et al.*, 2015; Johansson *et al.*, 2016).

Glyphosate (GLY) is a widely used herbicide in urban and rural environments (Gillezeau *et al.*, 2019). Since the mid-1990s, the introduction of GLY-resistant crops resulted in a dramatic increase in GLY agricultural usage for weed control (Benbrook, 2016). Commercially, GLY salts are mixed with surfactants to increase penetrative capacity in plants (Benbrook, 2016) and referred to as GLY-based herbicides (GBHs). Both GLY and GBHs are also broadly utilized for weed control in home gardens, railways, and roadways.

The presence of GLY residues in food stuffs (Bøhn *et al.*, 2014; Granby *et al.*, 2003; Zoller *et al.*, 2018, 2020) and in human urine (Lemke *et al.*, 2021; Mills *et al.*, 2017; Soukup *et al.*, 2020) supports human GLY exposure. A review of human GLY studies from Europe and the United States concluded lack of GLY health risk based upon the level of exposure being lower than the proposed acceptable daily intake established by the European Food Safety Authority (0.5 mg/kg/body weight) (Niemann *et al.*, 2015). The level of GLY and/or the main metabolite aminomethylphosphonic acid (AMPA) concentrations in human urine samples relative to known ingested concentrations determined that approximately 1%–6% of ingested GLY was recoverable in urine (Faniband *et al.*, 2021), whereas GLY is readily excreted through feces and urine (Williams *et al.*, 2000).

In rodents, GLY exposure increased atretic ovarian follicle number and decreased antral follicle number at doses of 126 or 315 mg/kg/day (Hamdaoui *et al.*, 2018). Increased granulosa and theca cell proliferation and decreased follicle-stimulating hormone receptor and growth and differentiation factor 9 mRNA level at a GLY dose of 2 mg/kg in lambs has been observed (Alarcón *et al.*, 2019). In contrast, no observable effects of 5 or 10 weeks of 2 mg/kg GLY exposure on follicle number or other ovarian endpoints have been reported in mice (Ganesan *et al.*, 2020). Further, a GLY dose response study determined no effect of 20 weeks of GLY (0.25–1.5 mg/kg) exposure on follicle number with the exception of the upper 2 mg/kg dose, in which increased follicle number relative to control treated mice was noted, in the absence of an effect on estrous cyclicity in mice (Ganesan and Keating, 2020). Exposure to 500 µM GLY impaired oocyte maturation through reduced germinal vesicle breakdown proposed to be due to increased reactive oxygen species generation (Zhang *et al.*, 2019). Interestingly, exposure to 0.25–2 mg/kg GLY altered ovarian proteins with mitochondrial and oxidative stress response functions (Ganesan and Keating, 2020). The F2 offspring of F1 rats exposed both perinatally and during lactation to GBH (2 or 200 mg/kg) had delayed fetal growth and congenital anomalies at the higher GBH dose (Milesi *et al.*, 2018). In humans, GLY urinary concentration of pregnant women was correlated with shortened gestation length (Lesseur *et al.*, 2022; Parvez *et al.*, 2018). Thus, there is rationale for consideration of GLY as a reproductive toxicant.

Based upon evidence of GLY-induced alterations to ovarian and reproductive endpoints and the recognized ability of toxicant exposures to have multi- and trans-generational effects (Milesi *et al.*, 2021; Patisaul, 2021), this study investigated the hypothesis that GLY exposure would affect ovarian endpoints in dams due to pre-conceptional exposure. An exposure level of 2 mg/kg/day was chosen, based upon lack of observable effects of this dose on reproductive and developmental endpoints by the Environmental Protection Agency (ATSDR, 2020). An exposure duration of 70 days was conducted to encompass an entire round of folliculogenesis (Hoyer, 2001) to evaluate if GLY exposure affected ovarian follicular composition and the maternal liver and ovary proteome.

## MATERIALS AND METHODS

###  

####  

##### Reagents

GLY (CAS no. 1071-83-6), Eosin Y, EDTA, paraformaldehyde (PFA), Tween20, 2-β-mercaptoethanol, Tris base, Tris HCL, and sodium chloride were purchased from Sigma-Aldrich Inc. (St Louis, Missouri). Mayer’s Hematoxylin (F380-2) was purchased from Rowley Biochemical Inc. (Danvers, Massachusetts). Sodium citrate, glycerol, citric acid, and Pierce BCA protein assay kit were from Thermo Fisher Scientific.

##### Animal dosing and breeding

All animal experiments were approved by the Institutional Animal Care and Use Committee at Iowa State University. Female C57Bl/6 mice were purchased from Jackson Laboratories aged 6 weeks. After 1 week of acclimation, mice were randomly split into 2 groups: one group received saline *per os* from a pipette tip as vehicle control (CT; *n* = 20) and the other group received GLY (2 mg/kg; *n* = 20) diluted in saline once per day for 10 weeks by the same route. Mice were maintained under controlled room temperature (21°C–22°C) and 12 h light: dark cycles with *ad libitum* access to food and water. Food intake and body weight were measured weekly. Food consumption was calculated as the mean chow (g) eaten per cage per week.

After 10 weeks of CT or GLY exposure, females were housed with C57Bl/6 males for approximately 5 days and the appearance of a vaginal plug was monitored daily. The monitoring of the vaginal plug observance was designated as gestational day 1 (GD1). The pregnancy success for each group was calculated as: the number of pregnant dams as a percent of the total number of females housed with males. At GD14, CT and GLY females (*n* = 10 per treatment) were euthanized for tissue collection and embryo enumeration and designated as GD14. The remainder of the pregnant dams (*n* = 10 per treatment) completed pregnancy and lactation and were euthanized 1-week post-weaning and designated as PW. All animals in the study were euthanized by CO_2_ asphyxiation followed by cervical dislocation.

##### Tissue collection

Liver, spleen, and ovaries were collected from GD14 and PW dams, weighed and the liver and ovary were immediately either snap frozen in liquid nitrogen or fixed in 4% PFA overnight at 4°C for histological analysis. One ovary was frozen and stored at −80°C for protein analysis, and another was fixed in 4% PFA for hematoxylin and eosin staining. Blood was collected by cardiac puncture, stored on ice and centrifuged for 15 min at 10 000 rpm for serum separation.

##### Serum 17β-estradiol and progesterone hormone level quantification

The level of 17β-estradiol and progesterone in serum was quantified by the Ligand Assay & Analysis Core of the Center for Research in Reproduction, University of Virginia with 2 technical replicates per sample.

##### Histological analysis

Ovaries were fixed in 4% PFA for 24 h, transferred to 70% ethanol, and then paraffin embedded. One ovary from each mouse was sectioned (5 μM thickness) and every 6th section was mounted and stained with hematoxylin and eosin. Oocyte-containing follicles and corpora lutea (CLs) were identified and counted in every 12th section. Healthy follicles were classified and counted as described previously (Flaws *et al.*, 1994). Atretic follicles were differentiated from healthy follicles by the appearance of pyknotic bodies and intense eosinophilic staining of oocytes.

##### LC-MS/MS analysis

Ovarian and liver tissue homogenates were analyzed by liquid chromatography tandem mass spectrometry (LC-MS/MS). Briefly, protein extracts were reduced with dithiothreitol, with the cysteine groups modified with iodoacetamide, and digested overnight with trypsin/Lys-C. The addition of formic acid stopped digestion before drying down samples in a SpeedVac. Samples were desalted using C18 MicroSpin Columns (Nest Group SEM SS18V) before drying again in a SpeedVac. Peptide retention time calibration (PRTC) standard (Pierce part no. 88320) was spiked into the sample to serve as an internal control. The peptides were then separated by liquid chromatography and analyzed by MS/MS by fragmenting each peptide using a Q Exactive Hybrid Quadrupole-Orbitrap Mass Spectrometer (Thermo Fisher Scientific). The resulting intact and fragmentation pattern was compared with a theoretical fragmentation pattern (from either MASCOT or Sequest HT) for protein identification. Label-free quantification technique used the Minora Feature Detector to detect and quantify isotopic clusters. The PRTC areas were used to normalize the data between samples. Proteins were identified by searching databases with both MS and MS/MS data using Mascot software as described previously (Xia and Wishart, 2016).

##### Statistical analysis

Statistical analysis was conducted by unpaired *t* test using Prism 9.0.1 software (GraphPad Prism). Statistical significance was defined as *p *<* *.05. For the pathway analyses based on LC-MS/MS results, proteins that differed from CT at *p *<* *.1 were included. Biological pathways altered by GLY exposure were predicted using DAVID v 6.8 software. The number for “Gene Found” represented the number of genes identified from proteomics analyses. The number for “Gene Pathway” represented the total number of genes in the particular pathway as found in the Kyoto Encyclopedia of Genes and Genomes (KEGG) pathway database. The false discovery rate (FDR) for every presented pathway was also calculated using DAVID v 6.8 software. The results in barcharts are represented mean ± standard error of mean (SEM).

##### Samples identification methods

The sample identity for follicle counting, 17β-estradiol, progesterone, and proteomic analyses was not known to the investigator conducting the analyses.

## RESULTS

###  

#### Influence of GLY Pre-Conceptional Exposure on Body Weight

**Figure 1.**
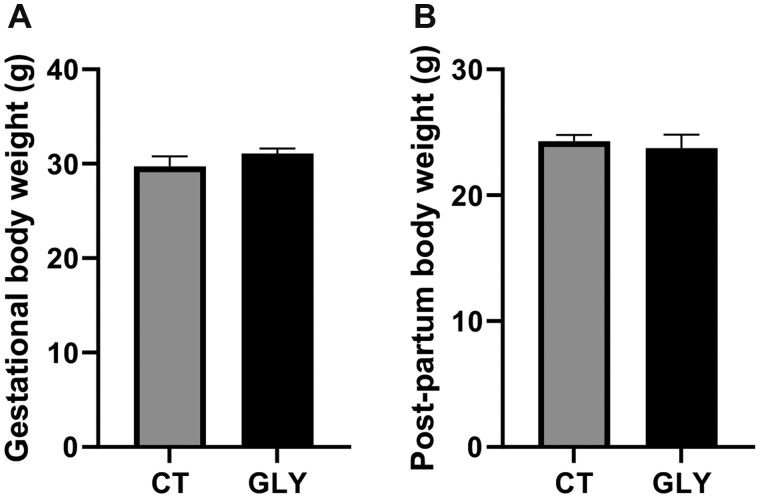
Impact of pre-conceptional GLY exposure on GD14 and PW female body weight. Female C57Bl/6 mice were exposed to saline vehicle control (CT) or GLY (2 mg/kg) pre-conceptionally for 10 weeks daily *per os.* A, GD14 dam weight (*n *= 7 for both CT and GLY). B, PW dam body weight (*n* = 8 for CT, *n* = 3 for GLY). Data points represent mean ± SEM.

The mean body weight for females at the end of 10 weeks of dosing before mating with males did not differ for GLY-exposed females (*p *>* *.05; CT = 20.25 ± 0.3 g; GLY = 21 ± 0.4 g). The food eaten per cage per week during the dosing period also did not differ between CT and GLY groups. Pre-conceptional GLY exposure did not affect body weight at GD14 (*p *>* *.05; CT = 29.07 ± 1.1 g; GLY = 31.07 ± 0.5 g; Figure 1A) or PW (*p *>* *.05; CT = 24.30 ± 0.5 g, GLY = 23.73 ± 1.1 g; Figure 1B).

#### Effect of Pre-Conceptional GLY Exposure on Pregnancy Success

Pregnancy success in CT mice was 75% with 15 out of 20 females conceiving and delivering offspring (data not shown). In GLY-exposed mice, pregnancy success was reduced to 55% with 11 of 20 females delivering offspring (data not shown).

#### Impact of Pre-Conceptional GLY Exposure on Organ Weight

**Figure 2.**
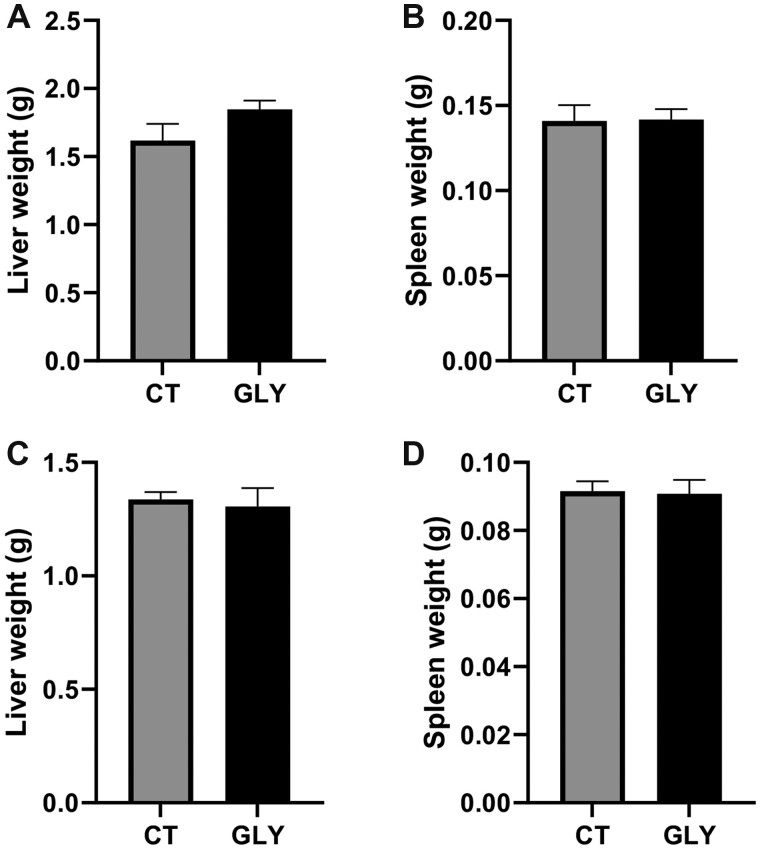
Impact of pre-conceptional GLY exposure on female organ weight. Female C57Bl/6 mice were exposed to saline vehicle control (CT) or GLY (2 mg/kg) pre-conceptionally for 10 weeks daily *per os*. (A) Liver and (B) spleen weight in GD14 dams (*n* = 7 for both CT and GLY). (C) Liver and (D) spleen weight in PW dams (*n* = 8 for CT, *n* = 3 for GLY). Data points represent mean ± SEM.

**Figure 3.**
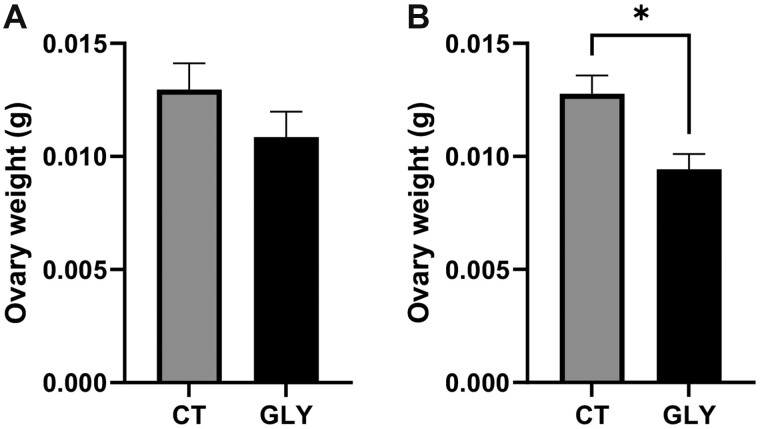
Impact of pre-conceptional GLY exposure on GD14 and PW ovarian weight. Female C57Bl/6 mice were exposed to saline vehicle control (CT) or GLY (2 mg/kg) pre-conceptionally for 10 weeks daily *per os*. Ovarian weight of (A) GD14 (*n* = 7 for both CT and GLY) and (B) PW dams (*n* = 8 CT, *n* = 3 for GLY). Data points represent mean ± SEM. Asterisk represents difference from CT; *p *<* *.05.

There was no difference (*p *>* *.05) in liver or spleen weight in GLY exposed relative to CT mice at GD14 (Figs. 2A and 2B) or PW (Figs. 2C and 2D). Ovarian weight did not differ between treatment at GD14 (*p *>* *.05; CT = 0.013 ± 0.001 g; GLY = 0.011 ± 0.001 g; Figure 3A). Ovarian weight was lower in GLY-exposed PW dams (*p *<* *.05; CT = 0.013 ± 0.0008 g; GLY = 0.009 ± 0.0007 g; Figure 3B).

#### Impact of Pre-Conceptional GLY Exposure on Circulating Serum 17β-Estradiol and Progesterone Levels

**Figure 4.**
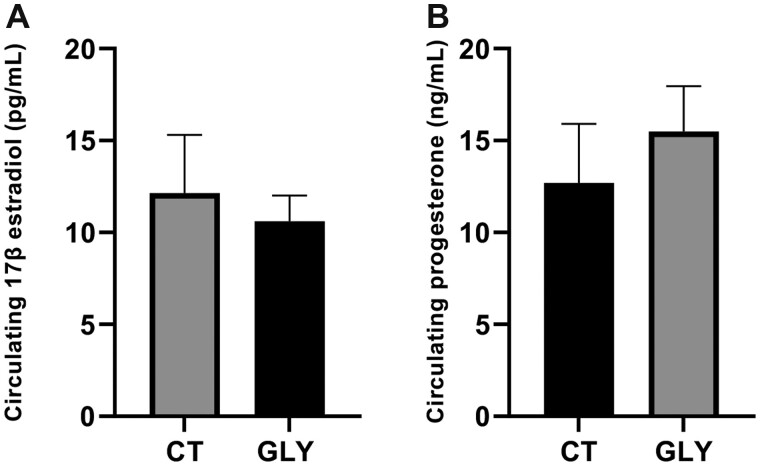
Impact of pre-conceptional GLY exposure on circulating 17β-estradiol and progesterone in GD14 dams. Female C57Bl/6 mice were exposed to saline vehicle control (CT) or GLY (2 mg/kg) pre-conceptionally for 10 weeks daily *per os*. (A) 17β-estradiol (*n* = 7 for both CT and GLY) and (B) progesterone level (*n* = 3 for CT, *n* = 5 for GLY). Data points represent mean ± SEM.

The levels of 17β-estradiol and progesterone were measured only in the serum obtained from the GD14 dams due to a low volume of serum obtained from PW dams. There was no treatment effect on serum 17β-estradiol (*p *<* *.05; CT = 12.14 ± 3.2 pg/ml; GLY = 10.61 ± 1.4 pg/ml; Figure 4A) or progesterone (*p *<* *.05; CT = 12.7 ± 3.2 ng/ml; GLY = 15.5 ± 2.5 ng/ml; Figure 4B) at GD14.

#### Effect of Pre-Conceptional GLY Exposure on Litter Size

**Figure 5.**
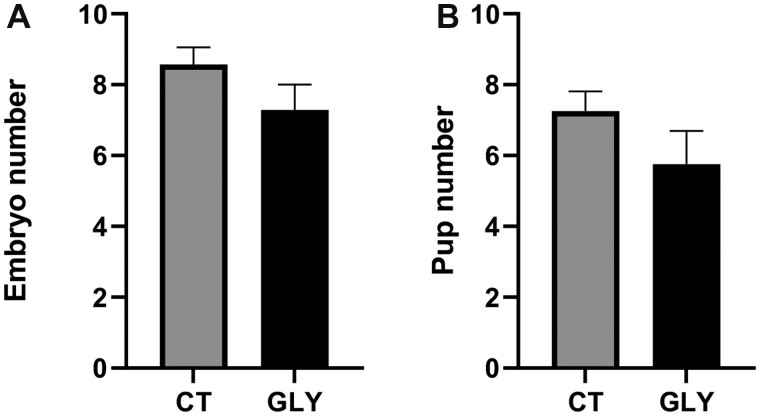
Effect of pre-conceptional GLY exposure on litter size. Female C57Bl/6 mice were exposed to saline vehicle control (CT) or GLY (2 mg/kg) pre-conceptionally for 10 weeks daily *per os*. A, The mean number of embryos per litter at GD14 (*n* = 7 for both CT and GLY). B, The mean number of pups born alive per litter (*n* = 8 for CT, *n *= 4 for GLY). *N* represents the litter number. Data points represent mean ± SEM.

The mean number of embryos per litter was similar for GD14 GLY exposed compared with CT dams (*p *>* *.05; CT = 8.6 ± 0.5; GLY = 7.3 ± 0.7; Figure 5A). The mean number of pups born alive per litter did not differ between CT and GLY exposed dams (*p *>* *.05; CT = 7.3 ± 0.6; GLY = 5.8 ± 0.9; Figure 5B).

#### Influence of Pre-Conceptional GLY Exposure on Ovarian Follicle Number and Classification

**Figure 6.**
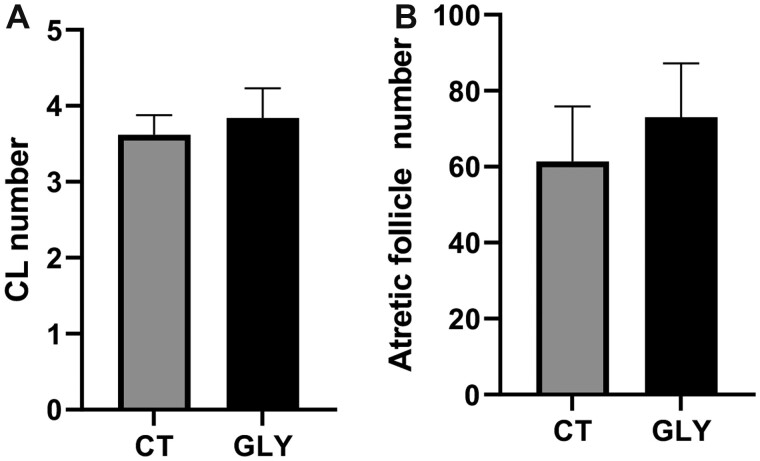
Effect of pre-conceptional GLY exposure on follicle number in GD14 dams. Female C57Bl/6 mice were exposed to saline vehicle control (CT) or GLY (2 mg/kg) pre-conceptionally for 10 weeks daily *per os*. A, The number of CL in the ovaries from GD14 dams (*n* = 5 for both CT and GLY). B, The number of atretic follicles in ovaries of GD14 dams (*n *= 5 for both CT and GLY). *N* represents the number of dams. Data points represent mean ± SEM.

**Figure 7.**
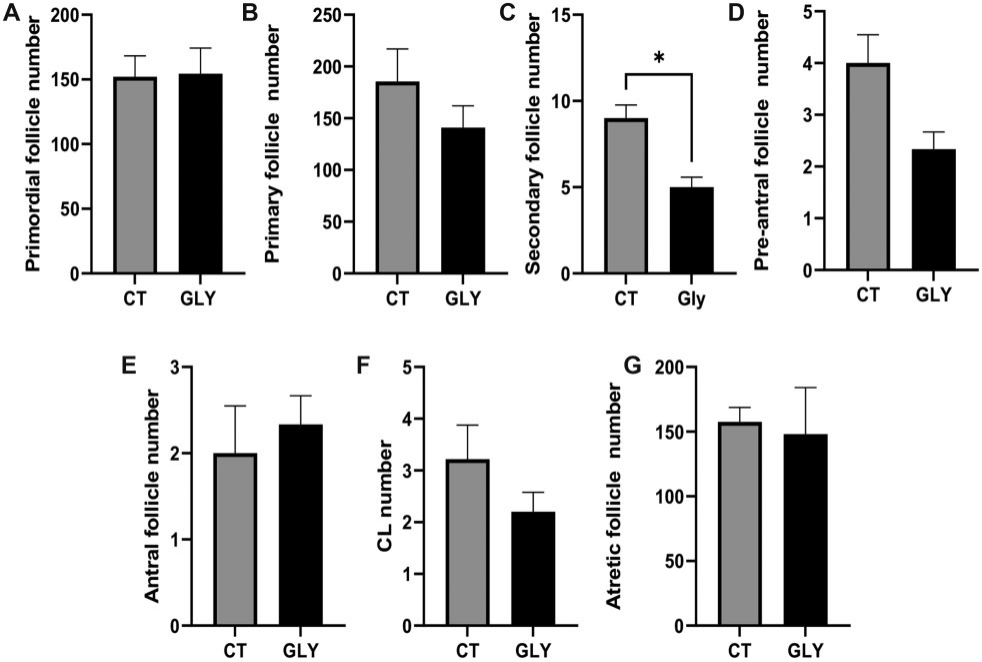
Effect of pre-conceptional GLY exposure on ovarian follicle number in PW dams. Female C57Bl/6 mice were exposed to saline vehicle control (CT) or GLY (2 mg/kg) pre-conceptionally for 10 weeks daily *per os*. (A) Primordial, (B) primary, (C), secondary, (D), pre-antral, and (E) antral follicle number, (F) CL number, and (G) atretic follicle number in PW dams (*n *= 5 for CT, and *n *= 3 for GLY). *N* represents the number of dams. Data points represent mean ± SEM. Asterisk represents difference from CT; *p *<* *.05.

The number of CL and atretic follicles were counted in GD14 ovaries. There was no effect of pre-conceptional GLY exposure on the number of CL (*p *>* *.05; CT = 3.6 ± 0.3; GLY = 3.8 ± 0.4; Figure 6A) or atretic follicles (*p *>* *.05; CT = 61.4 ± 14.5; GLY = 73.0 ± 14.1; Figure 6B). In PW dams, the number of primordial (Figure 7A), primary (Figure 7B), pre-antral (Figure 7D), antral (Figure 7E), CL (Figure 7F), and atretic follicles (Figure 7G) did not differ (*p *>* *.05) between CT and GLY exposed dams. The number of secondary follicles (Figure 7C) were lower (*p *<* *.05; CT = 9 ± 0.8; GLY = 5 ± 0.6) in the ovaries of GLY exposed relative to CT dams.

#### Effect of Pre-Conceptional GLY Exposure on GD14 Ovarian and Hepatic Proteome

**Figure 8.**
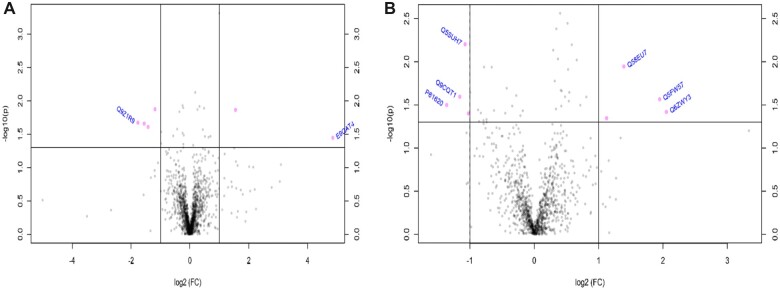
Impact of pre-conceptional GLY exposure on ovarian and hepatic proteome of GD14 dams. Female C57Bl/6 mice were exposed to saline vehicle control (CT) or GLY (2 mg/kg) pre-conceptionally for 10 weeks daily *per os*. (A) Ovary (*n* = 5 for CT, and *n *= 4 for GLY) and (B) liver (*n* = 5 for CT, and *n *= 4 for GLY) were collected at GD14 and proteins analyzed by LC-MS/MS. Bioinformatic analyses were performed to determine differences in protein abundance due to GLY exposure relative to CT. The pink dots in the volcano plots above the horizontal dotted line indicate proteins that differed in abundance with fold change > 2 and *p *<* *.05.

In GD14 mice, GLY exposure altered (*p *<* *.05) the abundance of 26 ovarian proteins, relative to CT mice (Table 1) with an FDR > 0.05. Out of those, the abundance of 2 proteins was increased and 4 proteins was decreased with a fold change > 2 in GLY compared with CT dams (Figure 8A). Protein transport protein sec16 (SEC16A) was increased 29-fold by GLY exposure. Pathway analyses of the ovarian altered proteins performed with DAVID did not identify possible targets with FDR < 0.1.

**Table 1.** Ovarian proteins altered by GLY exposure in GD14 dams

Protein name	log2(FC)	*p* value
Protein transport protein Sec16A	4.8591	.035915
Pyridoxal-dependent decarboxylase domain-containing protein 1	1.5537	.013687
Nup214 protein	1	.000487
ADP-ribose glycohydrolase MACROD1	0.69986	.033962
Nonspecific serine/threonine protein kinase	0.63402	.035074
YTH domain-containing family protein 3	0.5125	.034148
Eukaryotic translation initiation factor 1A, X-chromosomal	0.48301	.012895
40S ribosomal protein S21	0.4387	.027007
Receptor expression-enhancing protein	0.32646	.012106
Polyadenylate-binding protein 1	0.25679	.013489
Importin N-terminal domain-containing protein R	0.18224	.007486
Heat shock 70 kDa protein 4	0.11288	.046343
Thyroid hormone receptor-associated protein	−0.20908	.03274
Proteasome (Prosome, macropain) 28 subunit, alpha	−0.22259	.044676
Heterogeneous nuclear ribonucleoprotein	−0.22832	.012654
cAMP-dependent protein kinase type II-beta regulatory subunit	−0.24806	.030404
Hemopexin	−0.25051	.011695
Ribosomal protein L19	−0.35551	.014565
40S ribosomal protein S13	−0.43121	.026505
Tensin-3	−0.5652	.033498
Long-chain-fatty-acid–CoA ligase 1	−0.97356	.029265
Guanylate-binding protein 2	−0.97623	.043476
Histidine–tRNA ligase	−1.1861	.013373
MOB1, Mps one binder kinase activator-like 1A	−1.4189	.024608
Elongation factor 1-alpha 2	−1.5525	.021982
Protease, serine 1 (trypsin 1)	−1.7669	.021208

In the liver, GLY exposure altered (*p *<* *.05) the level of 41 proteins relative to CT mice (Table 2) with FDR > 0.05. A hepatic steroidogenic protein, 17-beta-hydroxysteroid dehydrogenase 13 (HSD17B13) was decreased by 0.6-fold. Proteins involved in xenobiotic metabolism were altered in livers of GLY-exposed dams and included glutathione *S*-transferase alpha (GSTA5; 1.6-fold increase), cytochrome P450 isoform 2C40 (CYP2C40; 0.8-fold decrease), sulfotransferase 1A1 (SULT1A1; 1.3-fold increase), cytochrome P450 isoform 2C37 (CYP2C37; 0.5-fold decrease), and cytochrome P450 isoform 2D10 (CYP2D10; 0.9-fold decrease; Table 2). Within the proteins that were altered by GLY exposure, hepatic protein abundance of 4 proteins increased and 4 decreased with a fold change >2 compared with CT dams (Figure 8B). Both 40S ribosomal protein S27-like (RPS27) and Glycine N-acyltransferase-like (GM4952) proteins were increased around 4-fold compared with CT dams.

**Table 2.** Hepatic proteins altered in GLY-exposed GD14 dams

Protein name	log2(FC)	*p* value
40S ribosomal protein S27-like	2.0531	.038138
Glycine N-acyltransferase-like protein	1.9495	.027228
Rbp1 protein	1.3924	.011364
Proteasome subunit beta type-5	1.125	.04518
Actin-related protein 2/3 complex subunit 4	0.8095	.025579
Glutathione transferase	0.71643	.031901
Serine/arginine-rich splicing factor 1	0.66019	.0095619
40S ribosomal protein S26	0.58371	.0063546
Calmodulin-2	0.54516	.022671
Cytochrome c oxidase subunit 7C, mitochondrial	0.53707	.028641
Signal recognition particle subunit SRP72	0.52603	.012295
40S ribosomal protein S28	0.51754	.0035913
Ribosomal protein S10	0.47892	.025479
Parkinson disease protein 7 homolog	0.42685	.021807
Sulfotransferase	0.42095	.032665
Ribosome-binding protein 1	0.4121	.021396
General vesicular transport factor p115	0.40249	.0027542
Glucosidase 2 subunit beta	0.36828	.0092784
Uncharacterized protein	0.34407	.0041649
Coatomer subunit alpha	0.3376	.035259
40S ribosomal protein S4	0.31858	.016734
Receptor of activated protein C kinase 1	0.29557	.0073505
Protein NDRG2	−0.17772	.037023
Cytochrome P450 2D10	−0.21536	.042145
Proteasome subunit alpha type	−0.27902	.024665
Cytochrome P450 2C40	−0.34197	.032644
Enoyl-CoA hydratase domain-containing protein 3, mitochondrial	−0.4281	.020302
Basigin	−0.44971	.039483
Sodium/bile acid cotransporter	−0.62562	.036768
Dihydropyrimidinase-related protein 2	−0.66024	.011562
26S proteasome non-ATPase regulatory subunit 5	−0.74082	.046609
17-beta-hydroxysteroid dehydrogenase	−0.78023	.011526
Rho GTPase-activating protein 29	−0.78841	.047922
Very-long-chain (3R)-3-hydroxyacyl-CoA dehydratase 3	−0.8298	.039349
Uncharacterized protein	−0.85731	.049258
MSP domain-containing protein	−0.87332	.036146
Cingulin	−0.99599	.022682
Cytochrome P450 2C37	−1.0215	.039765
Clathrin interactor 1	−1.0733	.0062831
Methylthioribose-1-phosphate isomerase	−1.1561	.025484
Protein transport protein Sec61 subunit alpha isoform 1	−1.3603	.031718

For the GD14 hepatic proteome changes induced by GLY exposure, enrichment analysis identified 10 pathways with FDR < 0.07: chemical carcinogenesis, linoleic acid metabolism, steroid hormone biosynthesis, retinol metabolism, arachidonic acid metabolism, ribosome, inflammatory mediator regulation of TRP channels, metabolic pathways, serotonergic synapse, and Parkinson’s disease (Table 3).

**Table 3.** KEGG pathways altered in liver of GLY-exposed GD14 dams

Pathway	Gene_Found	Gene_Pathway	FDR
Chemical carcinogenesis	9	84	5.94E-05
Linoleic acid metabolism	6	50	0.001856714
Steroid hormone biosynthesis	7	92	0.001856714
Retinol metabolism	7	97	0.001856714
Arachidonic acid metabolism	6	86	0.014565451
Ribosome	7	179	0.017561422
Inflammatory mediator regulation of TRP channels	6	127	0.045517365
Metabolic pathways	20	1572	0.045517365
Serotonergic synapse	6	131	0.046510328
Parkinson's disease	6	264	0.069861538

#### Impact of Pre-Conceptional GLY Exposure on the Hepatic Proteome of PW Dams

**Figure 9.**
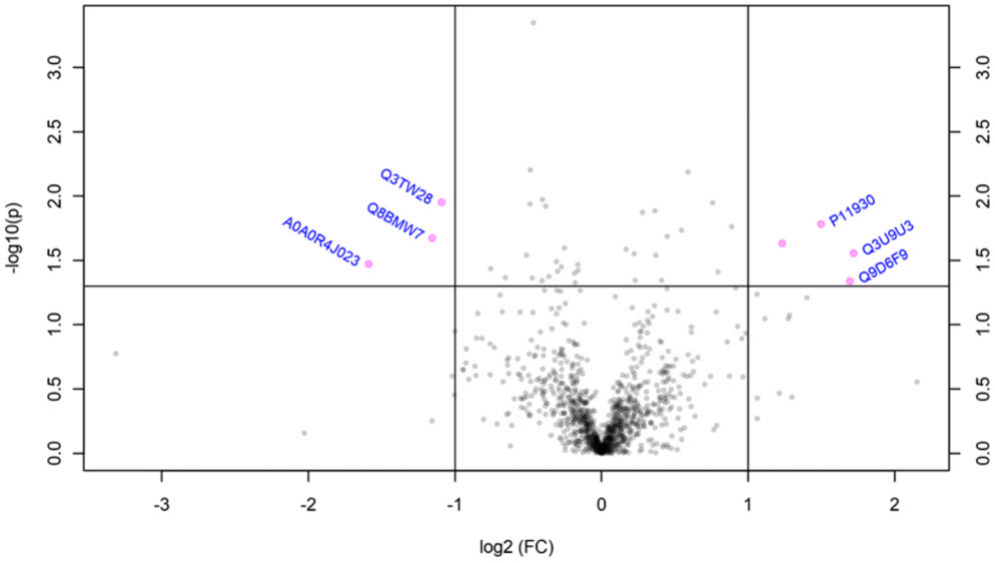
Impact of pre-conceptional GLY exposure on the hepatic proteome of PW dams. Female C57Bl/6 mice were exposed to saline vehicle control (CT) or GLY (2 mg/kg) pre-conceptionally for 10 weeks daily *per os*. Livers (*n* = 4 for CT, and *n* = 3 for GLY) were collected from PW dams and proteins analyzed by LC-MS/MS. Bioinformatic analyses were performed to determine differences in protein abundance due to GLY exposure relative to CT. Pink dots in volcano plots above the horizontal dotted line indicate proteins that differed in abundance with fold change > 2 and *p *<* *.05.

GLY-exposed PW dams had alterations (*p *<* *.05) to the abundance of 39 hepatic proteins relative to CT mice (Table 4; FDR > 0.05), including the xenobiotic biotransformation proteins cytochrome P450 isoform 2D22 (CYP2D22; 0.8-fold decrease) and dimethylaniline monooxygenase 1 (FMO1; 0.9-fold decrease). The abundance of 4 proteins were increased and 3 proteins were decreased by GLY exposure with a fold change > 2 (Figure 9). Pathway analyses of the hepatic proteome performed with DAVID platform did not identify targets with FDR < 0.1.

**Table 4.** Hepatic proteins altered in GLY-exposed PW dams

Protein name	log2(FC)	*p* value
Tubulin beta chain	1.7202	.027847
Tubulin beta-4A chain	1.6954	.045874
Nucleoside diphosphate-linked moiety X motif 19	1.4978	.016522
Coatomer subunit delta	1.2332	.023293
Eukaryotic translation initiation factor 4 gamma 1	0.88856	.017282
Calumenin	0.79541	.038917
Clustered mitochondria protein homolog	0.75834	.011274
Dimethyladenosine transferase 1, mitochondrial	0.59067	.0065102
Mitochondrial antiviral-signaling protein	0.54541	.018431
Elongation factor Tu, mitochondrial	0.44876	.020531
Inorganic pyrophosphatase 2, mitochondrial	0.41068	.04521
Lipoamide acyltransferase component of branched-chain alpha-keto acid dehydrogenase complex, mitochondrial	0.37084	.028903
Inorganic diphosphatase	0.36458	.013001
40S ribosomal protein S4	0.27893	.013402
AB hydrolase-1 domain-containing protein	0.23006	.045266
3-hydroxyisobutyryl-CoA hydrolase, mitochondrial	0.22328	.028083
Fructose-bisphosphate aldolase	0.1679	.025904
Delta-1-pyrroline-5-carboxylate dehydrogenase, mitochondrial	−0.16064	.037779
Cytochrome c oxidase subunit 5A, mitochondrial	−0.16111	.034132
Translationally-controlled tumor protein	−0.18	.047163
Dimethylaniline monooxygenase [N-oxide-forming] 1	−0.18419	.039827
Enoyl-CoA hydratase domain-containing protein 3, mitochondrial	−0.25261	.025276
Tyrosine 3-monooxygenase/tryptophan 5-monooxygenase activation protein, beta polypeptide	−0.27372	.035466
NADH dehydrogenase [ubiquinone] flavoprotein 1, mitochondrial	−0.29884	.038423
Cytochrome P450, family 2, subfamily d, polypeptide 22	−0.30752	.032319
Rap1A-retro1	−0.35868	.042217
Glycogen phosphorylase, liver form	−0.38031	.012002
Proteasome subunit alpha type	−0.40282	.010637
60S ribosomal protein L28	−0.40588	.045524
Dehydrogenase/reductase SDR family member 1	−0.46466	.00045012
High mobility group nucleosome-binding domain-containing protein 5	−0.47242	.043406
Apolipoprotein A-II	−0.48597	.0062553
Atlastin-3	−0.48807	.011552
Persulfide dioxygenase ETHE1, mitochondrial	−0.5115	.028812
Predicted gene 884	−0.65678	.04312
Coronin	−0.75583	.036708
Tripeptidyl-peptidase 2	−1.0908	.011161
Magnesium transporter NIPA3	−1.155	.021219
Methylglutaconyl-CoA hydratase, mitochondrial	−1.589	.033778

## DISCUSSION

The potential risks of GLY chronic pre-conceptional exposure on ovarian follicle composition and the proteome of the ovary and liver both during and after pregnancy was investigated in this study. GLY’s widespread agricultural usage became possible due to development of GLY-resistant crops in the early 1990s (Benbrook, 2016). By 2014, approximately 240 million pounds were sprayed annually on U.S. agricultural fields (Benbrook, 2016). Excretion of GLY and its main metabolite, AMPA, have been detected in human urine (Connolly *et al.*, 2018; Curwin *et al.*, 2007; Faniband *et al.*, 2021; Knudsen *et al.*, 2017). In commercial formulations, GLY is mixed with surfactants to increase its penetrative qualities to inhibit weed growth (Mesnage *et al.*, 2019). The reproductive risks of GBHs have been assessed in human (Arbuckle *et al.*, 2001; Bell *et al.*, 2001; Farr *et al.*, 2004; Garry *et al.*, 2002; Savitz *et al.*, 1997), rodent (Dallegrave *et al.*, 2003; Guerrero Schimpf *et al.*, 2017; Kubsad *et al.*, 2019; Lorenz *et al.*, 2019, 2020; Ren *et al.*, 2018), and ruminant (Alarcón *et al.*, 2019) studies, though the dosage of GLY or GBHs varies widely between studies. Lack of overt ovotoxicity of GLY at a concentration of 2 mg/kg for 5 or 10 weeks of exposure has been reported (Ganesan *et al.*, 2020). However, the same dose impacted the ovarian proteome after a longer exposure paradigm of 20 weeks with particular effects on mitochondrial and oxidative stress proteins. Additionally, a higher number of ovarian follicles present within the ovary were noted implicating the potential of GLY to affect ovarian function (Ganesan and Keating, 2020). Whether these impacts resulted in fertility consequences remain unclear.

In this study, the effects of a chronic pre-conceptional exposure of GLY on maternal ovarian follicle number and the ovarian and hepatic proteome were investigated. The administration route chosen mimicked both oral and chronic exposure. The dose was chosen based upon previous studies by our group and others (Alarcón *et al.*, 2019; Ganesan and Keating, 2020; Ganesan *et al.*, 2020) and the duration of 70 days encompassed an entire round of folliculogenesis (Butcher *et al.*, 1974; Rajkovic *et al.*, 2006).

Though previous studies report a negative impact of GLY exposure on body weight (Ait Bali *et al.*, 2017; Jasper *et al.*, 2012; Yousef *et al.*, 1995), a difference in the weight gain between CT and GLY exposed mice after 10 weeks of exposure was not demonstrated. A similar lack of an effect of 2 mg/kg GLY on female body weight after 5 or 10 weeks of exposure was also shown in our previous studies (Ganesan *et al.*, 2020). Additionally, there was no impact of GLY exposure on food intake, with the caveat that the food intake measurements were per cage rather than total caloric intake per individual mouse. Thus, no evidence of an impact of glyphosate exposure on food consumption or weight gain was observed.

A reduction in pregnancy was noted due to GLY exposure. The mice were approximately 17 weeks of age at the time of breeding, thus, this age could influence pregnancy outcomes independent of chemical exposure. Although difficult to conclude if pregnancy loss was increased from this study design, a negative impact on uterine morphology and decidualization was reported previously in female rats that were exposed to 2 mg/kg of GBH neonatally (Ingaramo *et al.*, 2016), potentially affecting pregnancy success. An association between urinary GLY level and shortened gestation (Lesseur *et al.*, 2022; Parvez *et al.*, 2018) and oxidative stress (Eaton *et al.*, 2022) has also been reported in women. Previous studies reported a GLY impact on maternal weight (Ren *et al.*, 2018; Teleken *et al.*, 2020), however this was not noted in the current study in either the mid-gestation or post-partum mice. There was also no impact of GLY on liver or spleen weight. Interestingly, ovarian weight was reduced in PW mice due to pre-conceptional GLY exposure but this effect was not observed in GD14 mice. These findings could indicate an effect of GLY on ovarian composition intensified by ovarian aging and longer term studies are warranted.

The ovarian and placental hormones progesterone and 17β-estradiol are important for maintaining pregnancy, and dysregulation of their levels could result in a spectrum of pregnancy complications including miscarriage (Berkane *et al.*, 2017; Kumar and Magon, 2012). Several previous studies demonstrated the potential of GBHs to act as endocrine disruptors impacting the circulating levels of the progesterone and/or 17β-estradiol (Perego *et al.*, 2017; Ren *et al.*, 2018; Wrobel, 2018). However, previous studies from our group did not observe changes in estrous cyclicity or in circulating levels of 17β-estradiol or progesterone in female mice after 10 and/or 20 weeks of 2 mg/kg of GLY exposure (Ganesan and Keating, 2020). In the current study, circulating levels of 17β-estradiol and progesterone were similar between treatments in pregnant dams in contrast with studies documenting 17β-estradiol and progesterone effects during pregnancy when exposed to higher concentrations of GBHs (Ren *et al.*, 2018). Thus, pre-conceptional GLY exposure did not result in observable changes to 17β-estradiol and progesterone during gestation in this paradigm.

Our previous studies on the impact of GLY exposure at 2 mg/kg for 5 or 10 weeks on ovarian follicle number did not reveal changes in the number of follicles at any stage of development compared with control treated females (Ganesan *et al.*, 2020). However, after 20 weeks of the identical chronic GLY exposure as in the current study, females had a greater number of ovarian primordial and antral follicles compared with control mice (Ganesan and Keating, 2020). We did not observe a difference in the number of CL or atretic follicles in pre-conceptional GLY-exposed GD14 compared with CT mice. Contradictory to our findings, mice exposed to GLY dissolved in drinking water (0.5%) had higher numbers of atretic follicles (Ren *et al.*, 2018). In PW dams, decreased secondary follicle number was observed due to GLY exposure, which may be explanatory for the reduced ovarian weight observed. These divergent GLY effects are likely explained by difference in dosing paradigms between studies.

To identify modes of action of pre-conceptional GLY exposure on the ovary and liver, a non-targeted approach was taken and whole ovarian or hepatic protein was analyzed by LC-MS/MS. The impact of GLY on the maternal GD14 ovary included changes to 26 proteins with the abundance of SEC16A being increased by 29-fold. SEC16A is critical in its regulatory role of several proteins’ endoplasmic reticulum conventional and unconventional secretion (Piao *et al.*, 2017; Supek *et al.*, 2002; Watson *et al.*, 2006), and is involved in nutrient-induced autophagy (Zahoor and Farhan, 2018). Such a dramatic increase due to GLY exposure in pregnancy could be a sign of dysregulation of metabolic processes. The level of ADP-ribose glycohydrolase MACROD1, that participates in post-translational modifications of target proteins, was increased 1.6-fold in the ovaries of GD14 dams exposed to GLY. This protein is activated in response to environmental triggers (Feijs *et al.*, 2013) and involved in DNA damage response mechanisms (Yang *et al.*, 2020). Expressed in many tissues, MACROD1 abundance is elevated in several types of cancers (Feijs *et al.*, 2020). In addition, among the proteins demonstrating increased abundance in the ovarian tissues from the pregnant dams exposed to GLY, heat shock 70 kDa protein 4 (HSPA4) was increased (1.1-fold). Increased activity of HSPA4 and other heat shock proteins of the HSP110 family was implicated in carcinogenesis and drug resistance (Gotoh *et al.*, 2004; Kimura *et al.*, 2016). The increase in abundance of these proteins in the ovaries from GLY-exposed dams could indicate that, even at low concentrations, ovarian molecular changes are observed due to pre-conceptional GLY exposure. It is important to note that these findings represent a snapshot of the ovarian impacts and whether these changes could influence gestational outcomes or long-term fertility cannot be concluded.

There were changes to the abundance of 41 and 39 hepatic proteins in the GD14 and PW livers, respectively. The liver, as an important metabolic organ, serves as the major site for xenobiotic biotransformation with dramatically higher levels of Phase I and Phase II biotransformation enzymes than other organs (Gu and Manautou, 2012). This study identified a pre-conceptional GLY impact on the level of maternal hepatic xenobiotic Phase I biotransformation enzymes including CYP2C40, CYP2C37, and CYP2D10, and the Phase II biotransformation proteins SULT1A1 and GSTA5. In the livers of PW mice, two Phase I biotransformation enzymes, CYP2D22 and FMO1, were decreased due to GLY exposure. Interestingly, in both GD14 and PW dams, hepatic 40S ribosomal protein S4 (RPS4) level was increased and Enoyl-CoA hydratase domain-containing protein 3, mitochondrial (ECHDC3) abundance was decreased. This similar pattern of RPS4 and ECHDC3 proteins at both GD14 and PW may indicate a persistent influence of GLY exposure.

Hepatic proteomic pathway analyses demonstrated that pre-conceptional GLY exposure changed proteins that represented specific pathways during pregnancy. Among the potential pathway targeted by GLY at GD14 with FDR < 0.07 were chemical carcinogenesis, steroid hormone biosynthesis, retinol metabolism, and metabolic pathways, all of which are important for maintaining pregnancy.

Taken together, lack of observed impacts of GLY exposure on body weight, liver or spleen weight, food intake, GD14 ovary weight, GD14 ovarian follicle composition, GD14 17β-estradiol and progesterone levels suggest an absence of endocrine disruption and overt toxicity. Reduced PW secondary follicle number and ovarian weight and an altered ovarian and hepatic proteome suggest that pre-conceptional chronic GLY exposure may affect maternal ovarian and hepatic function. Further studies to evaluate any long term impacts of the changes observed herein are needed.

## FUNDING

National Institute of Environmental Health Sciences (R21ES026282 to A.F.K.).

## DECLARATION OF CONFLICTING INTERESTS

The authors declared no potential conflicts of interest with respect to the research, authorship, and/or publication of this article.

## References

[kfac098-B1] Ait Bali Y. , Ba-Mhamed S., Bennis M. (2017). Behavioral and immunohistochemical study of the effects of subchronic and chronic exposure to glyphosate in mice. Front. Behav. Neurosci. 11, 146. 28848410 10.3389/fnbeh.2017.00146 PMC5550406

[kfac098-B2] Alarcón R. , Ingaramo P. I., Rivera O. E., Dioguardi G. H., Repetti M. R., Demonte L. D., Milesi M. M., Varayoud J., Muñoz-de-Toro M., Luque E. H. (2019). Neonatal exposure to a glyphosate-based herbicide alters the histofunctional differentiation of the ovaries and uterus in lambs. Mol. Cell. Endocrinol. 482, 45–56. 30550814 10.1016/j.mce.2018.12.007

[kfac098-B3] Arbuckle T. E. , Lin Z., Mery L. S. (2001). An exploratory analysis of the effect of pesticide exposure on the risk of spontaneous abortion in an Ontario farm population. Environ. Health Perspect. 109, 851–857. 11564623 10.1289/ehp.01109851 PMC1240415

[kfac098-B4] ATSDR. (2020). In Toxicological Profile for Glyphosate (Services USDoHaH, Ed.), pp. 1–237. U.S. Department of Health and Human Services, Public Health Service, Atlanta, GA.

[kfac098-B5] Bell E. M. , Hertz-Picciotto I., Beaumont J. J. (2001). A case-control study of pesticides and fetal death due to congenital anomalies. Epidemiology 12, 148–156. 11246574 10.1097/00001648-200103000-00005

[kfac098-B6] Benbrook C. M. (2016). Trends in glyphosate herbicide use in the United States and globally. Environ. Sci. Eur. 28, 3. 27752438 10.1186/s12302-016-0070-0 PMC5044953

[kfac098-B7] Berkane N. , Liere P., Oudinet J.-P., Hertig A., Lefèvre G., Pluchino N., Schumacher M., Chabbert-Buffet N. (2017). From pregnancy to preeclampsia: a key role for estrogens. Endocr. Rev. 38, 123–144. 28323944 10.1210/er.2016-1065

[kfac098-B8] Bøhn T. , Cuhra M., Traavik T., Sanden M., Fagan J., Primicerio R. (2014). Compositional differences in soybeans on the market: glyphosate accumulates in roundup ready gm soybeans. Food Chem. 153, 207–215. 24491722 10.1016/j.foodchem.2013.12.054

[kfac098-B9] Borman S. M. , Christian P. J., Sipes I. G., Hoyer P. B. (2000). Ovotoxicity in female Fischer rats and B6 mice induced by low-dose exposure to three polycyclic aromatic hydrocarbons: comparison through calculation of an ovotoxic index. Toxicol. Appl. Pharmacol. 167, 191–198. 10986010 10.1006/taap.2000.9006

[kfac098-B10] Brieño-Enríquez M. A. , García-López J., Cárdenas D. B., Guibert S., Cleroux E., Děd L., Hourcade J., Pěknicová J., Weber M., Del Mazo J. (2015). Exposure to endocrine disruptor induces transgenerational epigenetic deregulation of microRNAs in primordial germ cells. PLoS One 10, e0124296. 25897752 10.1371/journal.pone.0124296 PMC4405367

[kfac098-B11] Butcher R. L. , Collins W. E., Fugo N. W. (1974). Plasma concentration of LH, FSH, prolactin, progesterone and estradiol-17beta throughout the 4-day estrous cycle of the rat. Endocrinology 94, 1704–1708. 4857496 10.1210/endo-94-6-1704

[kfac098-B12] Connolly A. , Basinas I., Jones K., Galea K. S., Kenny L., McGowan P., Coggins M. A. (2018). Characterising glyphosate exposures among amenity horticulturists using multiple spot urine samples. Int. J. Hyg. Environ. Health. 221, 1012–1022. 30078418 10.1016/j.ijheh.2018.06.007

[kfac098-B13] Curwin B. D. , Hein M. J., Sanderson W. T., Striley C., Heederik D., Kromhout H., Reynolds S. J., Alavanja M. C. (2007). Pesticide dose estimates for children of Iowa farmers and non-farmers. Environ. Res. 105, 307–315. 17659274 10.1016/j.envres.2007.06.001

[kfac098-B14] Dallegrave E. , Mantese F. D., Coelho R. S., Pereira J. D., Dalsenter P. R., Langeloh A. (2003). The teratogenic potential of the herbicide glyphosate-roundup in Wistar rats. Toxicol. Lett. 142, 45–52. 12765238 10.1016/s0378-4274(02)00483-6

[kfac098-B15] Eaton J. L. , Cathey A. L., Fernandez J. A., Watkins D. J., Silver M. K., Milne G. L., Velez-Vega C., Rosario Z., Cordero J., Alshawabkeh A., et al (2022). The association between urinary glyphosate and aminomethyl phosphonic acid with biomarkers of oxidative stress among pregnant women in the protect birth cohort study. Ecotoxicol. Environ. Saf. 233, 113300. 35158254 10.1016/j.ecoenv.2022.113300 PMC8920761

[kfac098-B16] Faniband M. H. , Norén E., Littorin M., Lindh C. H. (2021). Human experimental exposure to glyphosate and biomonitoring of young Swedish adults. Int. J. Hyg. Environ. Health. 231, 113657. 33130428 10.1016/j.ijheh.2020.113657

[kfac098-B17] Farr S. L. , Cooper G. S., Cai J., Savitz D. A., Sandler D. P. (2004). Pesticide use and menstrual cycle characteristics among premenopausal women in the agricultural health study. Am. J. Epidemiol. 160, 1194–1204. 15583372 10.1093/aje/kwi006

[kfac098-B18] Feijs K. L. H. , Cooper C. D. O., Žaja R. (2020). The controversial roles of ADP-ribosyl hydrolases MACROD1, MACROD2 and TARG1 in carcinogenesis. Cancers (Basel) 12, 604. 32151005 10.3390/cancers12030604 PMC7139919

[kfac098-B19] Feijs K. L. H. , Verheugd P., Lüscher B. (2013). Expanding functions of intracellular resident mono-ADP-ribosylation in cell physiology. FEBS J. 280, 3519–3529. 23639026 10.1111/febs.12315

[kfac098-B20] Flaws J. A. , Doerr J. K., Sipes I. G., Hoyer P. B. (1994). Destruction of preantral follicles in adult rats by 4-vinyl-1-cyclohexene diepoxide. Reprod. Toxicol. 8, 509–514. 7881202 10.1016/0890-6238(94)90033-7

[kfac098-B21] Ganesan S. , Bhattacharya P., Keating A. F. (2013). 7,12-dimethylbenz[a]anthracene exposure induces the DNA repair response in neonatal rat ovaries. Toxicol. Appl. Pharmacol. 272, 690–696. 23969067 10.1016/j.taap.2013.08.013 PMC3885336

[kfac098-B22] Ganesan S. , Keating A. F. (2020). Ovarian mitochondrial and oxidative stress proteins are altered by glyphosate exposure in mice. Toxicol. Appl. Pharmacol. 402, 115116. 32634520 10.1016/j.taap.2020.115116 PMC8500330

[kfac098-B23] Ganesan S. , McGuire B. C., Keating A. F. (2020). Absence of glyphosate-induced effects on ovarian folliculogenesis and steroidogenesis. Reprod. Toxicol. 96, 156–164. 32592754 10.1016/j.reprotox.2020.06.011 PMC8500328

[kfac098-B24] Garry V. F. , Harkins M. E., Erickson L. L., Long-Simpson L. K., Holland S. E., Burroughs B. L. (2002). Birth defects, season of conception, and sex of children born to pesticide applicators living in the Red River Valley of Minnesota, USA. Environ. Health Perspect. 110(Suppl 3), 441–449. 12060842 10.1289/ehp.02110s3441 PMC1241196

[kfac098-B25] Gillezeau C. , van Gerwen M., Shaffer R. M., Rana I., Zhang L., Sheppard L., Taioli E. (2019). The evidence of human exposure to glyphosate: a review. Environ. Health 18, 2–2. 30612564 10.1186/s12940-018-0435-5 PMC6322310

[kfac098-B26] Gotoh K. , Nonoguchi K., Higashitsuji H., Kaneko Y., Sakurai T., Sumitomo Y., Itoh K., Subjeck J. R., Fujita J. (2004). Apg-2 has a chaperone-like activity similar to Hsp110 and is overexpressed in hepatocellular carcinomas. FEBS Lett. 560, 19–24. 14987991 10.1016/S0014-5793(04)00034-1

[kfac098-B27] Granby K. , Johannesen S., Vahl M. (2003). Analysis of glyphosate residues in cereals using liquid chromatography-mass spectrometry (LC-MS/MS). Food Addit. Contam. 20, 692–698. 13129785 10.1080/0265203031000109477

[kfac098-B28] Gu X. , Manautou J. E. (2012). Molecular mechanisms underlying chemical liver injury. Expert Rev. Mol. Med. 14, e4. 22306029 10.1017/S1462399411002110 PMC3704158

[kfac098-B29] Guerrero Schimpf M. , Milesi M. M., Ingaramo P. I., Luque E. H., Varayoud J. (2017). Neonatal exposure to a glyphosate based herbicide alters the development of the rat uterus. Toxicology 376, 2–14. 27287056 10.1016/j.tox.2016.06.004

[kfac098-B30] Hamdaoui L. , Naifar M., Rahmouni F., Harrabi B., Ayadi F., Sahnoun Z., Rebai T. (2018). Subchronic exposure to kalach 360 SL-induced endocrine disruption and ovary damage in female rats. Arch. Physiol. Biochem. 124, 27–34. 28708416 10.1080/13813455.2017.1352606

[kfac098-B31] Hatch E. E. , Troisi R., Wise L. A., Titus-Ernstoff L., Hyer M., Palmer J. R., Strohsnitter W. C., Robboy S. J., Anderson D., Kaufman R., et al (2011). Preterm birth, fetal growth, and age at menarche among women exposed prenatally to diethylstilbestrol (DES). Reprod. Toxicol. 31, 151–157. 21130156 10.1016/j.reprotox.2010.11.006 PMC3057340

[kfac098-B32] Hoyer P. B. (2001). Reproductive toxicology: current and future directions. Biochem. Pharmacol. 62, 1557–1564. 11755108 10.1016/s0006-2952(01)00814-0

[kfac098-B33] Hoyer P. B. , Keating A. F. (2014). Xenobiotic effects in the ovary: temporary versus permanent infertility. Expert Opin. Drug Metab. Toxicol. 10, 511–523. 24450963 10.1517/17425255.2014.880690

[kfac098-B34] Ingaramo P. I. , Varayoud J., Milesi M. M., Schimpf M. G., Muñoz-de-Toro M., Luque E. H. (2016). Effects of neonatal exposure to a glyphosate-based herbicide on female rat reproduction. Reproduction 152, 403–415. 27486271 10.1530/REP-16-0171

[kfac098-B35] Jasper R. , Locatelli G. O., Pilati C., Locatelli C. (2012). Evaluation of biochemical, hematological and oxidative parameters in mice exposed to the herbicide glyphosate-roundup(^®^). Interdiscip. Toxicol. 5, 133–140. 23554553 10.2478/v10102-012-0022-5 PMC3600513

[kfac098-B36] Johansson H. K. L. , Jacobsen P. R., Hass U., Svingen T., Vinggaard A. M., Isling L. K., Axelstad M., Christiansen S., Boberg J. (2016). Perinatal exposure to mixtures of endocrine disrupting chemicals reduces female rat follicle reserves and accelerates reproductive aging. Reprod. Toxicol. 61, 186–194. 27049580 10.1016/j.reprotox.2016.03.045

[kfac098-B37] Keating A. F. , Fernandez S. M., Mark-Kappeler C. J., Sen N., Sipes I. G., Hoyer P. B. (2011). Inhibition of pik3 signaling pathway members by the ovotoxicant 4-vinylcyclohexene diepoxide in rats. Biol. Reprod. 84, 743–751. 21076081 10.1095/biolreprod.110.087650 PMC3062039

[kfac098-B38] Kimura A. , Ogata K., Altan B., Yokobori T., Ide M., Mochiki E., Toyomasu Y., Kogure N., Yanoma T., Suzuki M., et al (2016). Nuclear heat shock protein 110 expression is associated with poor prognosis and chemotherapy resistance in gastric cancer. Oncotarget 7, 18415–18423. 26943774 10.18632/oncotarget.7821 PMC4951298

[kfac098-B39] Knudsen L. E. , Hansen P. W., Mizrak S., Hansen H. K., Morck T. A., Nielsen F., Siersma V., Mathiesen L. (2017). Biomonitoring of Danish school children and mothers including biomarkers of PBDE and glyphosate. Rev. Environ. Health. 32, 279–290. 28306542 10.1515/reveh-2016-0067

[kfac098-B40] Krarup T. (1969). Oocyte destruction and ovarian tumorigenesis after direct application of a chemical carcinogen (9:10-dimethyl-1:2-benzanthrene) to the mouse ovary. Int. J. Cancer. 4, 61–75. 4310334 10.1002/ijc.2910040109

[kfac098-B41] Kubsad D. , Nilsson E. E., King S. E., Sadler-Riggleman I., Beck D., Skinner M. K. (2019). Assessment of glyphosate induced epigenetic transgenerational inheritance of pathologies and sperm epimutations: generational toxicology. Sci. Rep. 9, 6372. 31011160 10.1038/s41598-019-42860-0 PMC6476885

[kfac098-B42] Kumar P. , Magon N. (2012). Hormones in pregnancy. Niger. Med. J. 53, 179–183. 23661874 10.4103/0300-1652.107549 PMC3640235

[kfac098-B43] Lemke N. , Murawski A., Schmied-Tobies M. I. H., Rucic E., Hoppe H.-W., Conrad A., Kolossa-Gehring M. (2021). Glyphosate and aminomethylphosphonic acid (AMPA) in urine of children and adolescents in Germany—human biomonitoring results of the German Environmental Survey 2014–2017 (GerES V). Environ. Int. 156, 106769. 34274860 10.1016/j.envint.2021.106769

[kfac098-B44] Lesseur C. , Pathak K. V., Pirrotte P., Martinez M. N., Ferguson K. K., Barrett E. S., Nguyen R. H. N., Sathyanarayana S., Mandrioli D., Swan S. H., et al (2022). Urinary glyphosate concentration in pregnant women in relation to length of gestation. Environ. Res. 203, 111811. 34339697 10.1016/j.envres.2021.111811 PMC8616796

[kfac098-B45] Lorenz V. , Milesi M. M., Schimpf M. G., Luque E. H., Varayoud J. (2019). Epigenetic disruption of estrogen receptor alpha is induced by a glyphosate-based herbicide in the preimplantation uterus of rats. Mol. Cell. Endocrinol. 480, 133–141. 30391669 10.1016/j.mce.2018.10.022

[kfac098-B46] Lorenz V. , Pacini G., Luque E. H., Varayoud J., Milesi M. M. (2020). Perinatal exposure to glyphosate or a glyphosate-based formulation disrupts hormonal and uterine milieu during the receptive state in rats. Food Chem. Toxicol. 143, 111560. 32640336 10.1016/j.fct.2020.111560

[kfac098-B47] Mattison D. , Schulman J. (1980). How xenobiotic chemicals can destroy oocytes. Contemp Obstet Gynecol 15, 157.

[kfac098-B48] Mesnage R. , Benbrook C., Antoniou M. N. (2019). Insight into the confusion over surfactant co-formulants in glyphosate-based herbicides. Food Chem. Toxicol. 128, 137–145. 30951798 10.1016/j.fct.2019.03.053

[kfac098-B49] Milesi M. M. , Lorenz V., Durando M., Rossetti M. F., Varayoud J. (2021). Glyphosate herbicide: reproductive outcomes and multigenerational effects. Front. Endocrinol. (Lausanne) 12, 672532. 34305812 10.3389/fendo.2021.672532 PMC8293380

[kfac098-B50] Milesi M. M. , Lorenz V., Pacini G., Repetti M. R., Demonte L. D., Varayoud J., Luque E. H. (2018). Perinatal exposure to a glyphosate-based herbicide impairs female reproductive outcomes and induces second-generation adverse effects in Wistar rats. Arch. Toxicol. 92, 2629–2643. 29947892 10.1007/s00204-018-2236-6

[kfac098-B51] Mills P. J. , Kania-Korwel I., Fagan J., McEvoy L. K., Laughlin G. A., Barrett-Connor E. (2017). Excretion of the herbicide glyphosate in older adults between 1993 and 2016. JAMA 318, 1610–1611. 29067413 10.1001/jama.2017.11726 PMC5818803

[kfac098-B52] Niemann L. , Sieke C., Pfeil R., Solecki R. (2015). A critical review of glyphosate findings in human urine samples and comparison with the exposure of operators and consumers. J. Verbr. Lebensm. 10, 3–12.

[kfac098-B53] Özen S. , Darcan Ş. Ş. (2011). Effects of environmental endocrine disruptors on pubertal development. J. Clin. Res. Pediatr. Endocrinol. 3, 1–6. 21448326 10.4274/jcrpe.v3i1.01 PMC3065309

[kfac098-B54] Parvez S. , Gerona R. R., Proctor C., Friesen M., Ashby J. L., Reiter J. L., Lui Z., Winchester P. D. (2018). Glyphosate exposure in pregnancy and shortened gestational length: a prospective Indiana birth cohort study. Environ. Health 17, 23. 29519238 10.1186/s12940-018-0367-0 PMC5844093

[kfac098-B23249844] Patisaul H. B. (2021). REPRODUCTIVE TOXICOLOGY: Endocrine disruption and reproductive disorders: impacts on sexually dimorphic neuroendocrine pathways. *Reproduction* 162, F111–F130. 10.1530/REP-20-0596 PMC8484365 33929341

[kfac098-B55] Perego M. C. , Schutz L. F., Caloni F., Cortinovis C., Albonico M., Spicer L. J. (2017). Evidence for direct effects of glyphosate on ovarian function: glyphosate influences steroidogenesis and proliferation of bovine granulosa but not theca cells in vitro. J. Appl. Toxicol. 37, 692–698. 27917511 10.1002/jat.3417

[kfac098-B56] Piao H. , Kim J., Noh S. H., Kweon H.-S., Kim J. Y., Lee M. G. (2017). Sec16A is critical for both conventional and unconventional secretion of CFTR. Sci. Rep. 7, 39887. 28067262 10.1038/srep39887 PMC5220342

[kfac098-B57] Rajkovic A. , Pangas S., Matzuk M. (2006). Follicular development: mouse, sheep, and human models. Knobil Neill Physiol. Reprod. 1, 383–423.

[kfac098-B58] Ren X. , Li R., Liu J., Huang K., Wu S., Li Y., Li C. (2018). Effects of glyphosate on the ovarian function of pregnant mice, the secretion of hormones and the sex ratio of their fetuses. Environ. Pollut. 243(Pt B), 833–841. 30245445 10.1016/j.envpol.2018.09.049

[kfac098-B59] Savitz D. A. , Arbuckle T., Kaczor D., Curtis K. M. (1997). Male pesticide exposure and pregnancy outcome. Am. J. Epidemiol. 146, 1025–1036. 9420527 10.1093/oxfordjournals.aje.a009231

[kfac098-B60] Soukup S. T. , Merz B., Bub A., Hoffmann I., Watzl B., Steinberg P., Kulling S. E. (2020). Glyphosate and AMPA levels in human urine samples and their correlation with food consumption: results of the cross-sectional KarMen study in Germany. Arch. Toxicol. 94, 1575–1584. 32232512 10.1007/s00204-020-02704-7 PMC7261737

[kfac098-B61] Supek F. , Madden D. T., Hamamoto S., Orci L., Schekman R. (2002). Sec16p potentiates the action of COPII proteins to bud transport vesicles. J. Cell Biol. 158, 1029–1038. 12235121 10.1083/jcb.200207053 PMC2173217

[kfac098-B62] Teleken J. L. , Gomes E. C. Z., Marmentini C., Moi M. B., Ribeiro R. A., Balbo S. L., Amorim E. M. P., Bonfleur M. L. (2020). Glyphosate-based herbicide exposure during pregnancy and lactation malprograms the male reproductive morphofunction in F1 offspring. J. Dev. Orig. Health Dis. 11, 146–153. 31309914 10.1017/S2040174419000382

[kfac098-B63] Watson P. , Townley A. K., Koka P., Palmer K. J., Stephens D. J. (2006). Sec16 defines endoplasmic reticulum exit sites and is required for secretory cargo export in mammalian cells. Traffic 7, 1678–1687. 17005010 10.1111/j.1600-0854.2006.00493.x PMC1761133

[kfac098-B64] Williams G. M. , Kroes R., Munro I. C. (2000). Safety evaluation and risk assessment of the herbicide roundup and its active ingredient, glyphosate, for humans. Regul. Toxicol. Pharmacol. 31, 117–165. 10854122 10.1006/rtph.1999.1371

[kfac098-B65] Wrobel M. H. (2018). Glyphosate affects the secretion of regulators of uterine contractions in cows while it does not directly impair the motoric function of myometrium in vitro. Toxicol. Appl. Pharmacol. 349, 55–61. 29705296 10.1016/j.taap.2018.04.031

[kfac098-B66] Xia J. , Wishart D. S. (2016). Using metaboanalyst 3.0 for comprehensive metabolomics data analysis. Curr. Protoc. Bioinformatics 55, 14.10.1–14.10.91. 10.1002/cpbi.11 27603023

[kfac098-B67] Yang R. , Wang Y. M., Zhang L., Zhao Z. M., Zhao J., Peng S. Q. (2016). Prepubertal exposure to T-2 toxin advances pubertal onset and development in female rats via promoting the onset of hypothalamic–pituitary–gonadal axis function. Hum. Exp. Toxicol. 35, 1276–1285. 26847343 10.1177/0960327116629529

[kfac098-B68] Yang X. , Ma Y., Li Y., Dong Y., Yu L. L., Wang H., Guo L., Wu C., Yu X., Liu X. (2020). Molecular basis for the MacroD1-mediated hydrolysis of ADP-ribosylation. DNA Repair (Amst) 94, 102899. 32683309 10.1016/j.dnarep.2020.102899 PMC7313891

[kfac098-B69] Yousef M. I. , Salem M. H., Ibrahim H. Z., Helmi S., Seehy M. A., Bertheussen K. (1995). Toxic effects of carbofuran and glyphosate on semen characteristics in rabbits. J. Environ. Sci. Health. B 30, 513–534. 7797819 10.1080/03601239509372951

[kfac098-B70] Zahoor M. , Farhan H. (2018). Crosstalk of autophagy and the secretory pathway and its role in diseases. Int. Rev. Cell Mol. Biol. **337**, 153–184. Oxford University Press 10.1016/bs.ircmb.2017.12.004 29551160

[kfac098-B71] Zhang J.-W. , Xu D.-Q., Feng X.-Z. (2019). The toxic effects and possible mechanisms of glyphosate on mouse oocytes. Chemosphere 237, 124435. 31352102 10.1016/j.chemosphere.2019.124435

[kfac098-B72] Zoller O. , Rhyn P., Rupp H., Zarn J. A., Geiser C. (2018). Glyphosate residues in Swiss market foods: monitoring and risk evaluation. Food Addit. Contam. Part B Surveill. 11, 83–91. 29284371 10.1080/19393210.2017.1419509

[kfac098-B73] Zoller O. , Rhyn P., Zarn J. A., Dudler V. (2020). Urine glyphosate level as a quantitative biomarker of oral exposure. Int. J. Hyg. Environ. Health. 228, 113526. 32305862 10.1016/j.ijheh.2020.113526

